# Refinement and validation of infrared thermal imaging (IRT): a non-invasive technique to measure disease activity in a mouse model of rheumatoid arthritis

**DOI:** 10.1186/s13075-020-02367-w

**Published:** 2020-11-30

**Authors:** Zeynab Nosrati, Marta Bergamo, Cristina Rodríguez-Rodríguez, Katayoun Saatchi, Urs O. Häfeli

**Affiliations:** 1 Faculty of Pharmaceutical Sciences, University of British Columbia, 2405 Wesbrook Mall, Vancouver, British Columbia V6T 1Z3 Canada; 2 Department of Physics and Astronomy, University of British Columbia, 6224 Agricultural Road, Vancouver, British Columbia V6T 1Z1 Canada; 3 Department of Pharmacy, Faculty of Health and Medical Sciences, University of Copenhagen, Universitetsparken 2, 2100 Copenhagen, Denmark

**Keywords:** Infrared thermal imaging, Animal model, Rheumatoid arthritis, Image processing, Preclinical study, Arthritis assessment

## Abstract

**Background:**

The discovery and development of new medicines requires high-throughput screening of possible therapeutics in a specific model of the disease. Infrared thermal imaging (IRT) is a modern assessment method with extensive clinical and preclinical applications. Employing IRT in longitudinal preclinical setting to monitor arthritis onset, disease activity and therapeutic efficacies requires a standardized framework to provide reproducible quantitative data as a precondition for clinical studies.

**Methods:**

Here, we established the accuracy and reliability of an inexpensive smartphone connected infrared (IR) camera against known temperature objects as well as certified blackbody calibration equipment. An easy to use protocol incorporating contactless image acquisition and computer-assisted data analysis was developed to detect disease-related temperature changes in a collagen-induced arthritis (CIA) mouse model and validated by comparison with two conventional methods, clinical arthritis scoring and paw thickness measurement. We implemented IRT to demonstrate the beneficial therapeutic effect of nanoparticle drug delivery versus free methotrexate (MTX) in vivo.

**Results:**

The calibrations revealed high accuracy and reliability of the IR camera for detecting temperature changes in the rheumatoid arthritis animal model. Significant positive correlation was found between temperature changes and paw thickness measurements as the disease progressed. IRT was found to be superior over the conventional techniques specially at early arthritis onset, when it is difficult to observe subclinical signs and measure structural changes.

**Conclusion:**

IRT proved to be a valid and unbiased method to detect temperature changes and quantify the degree of inflammation in a rapid and reproducible manner in longitudinal preclinical drug efficacy studies.

## Introduction

### Using animals as disease models in arthritis research

Animals have been used extensively in arthritis research, to examine the nature of the disease, develop new medicines, improve diagnostic procedures, and study drug toxicity. Animal research provides crucial data in order to assess whether a potential medicine will be effective and safe for use in humans [1]. In rheumatoid arthritis (RA) research, animal models are commonly used to screen the drug bioavailability at target inflamed joints, as well as to investigate drug disposition and therapeutic efficacy, thus providing information on the potential of new therapies. Therefore, the most important aspect of a model is its reproducibility, similarity, and relevance to the human disease being studied [2].

In preclinical drug efficacy studies, it is essential that the RA model shows similar arthritis onset, joint involvement pattern, as well as disease severity to humans, in order to accelerate drug screening and provide reliable data for subsequent testing in clinical trials.

RA models in rats and mice are most commonly induced by immunization with protein antigen, type II collagen, or homologous cartilage oligomeric matrix protein (COMP) leading to models of antigen-induced arthritis (AIA) [3–5], collagen-induced arthritis (CIA) [6], or COMP-induced arthritis [7], respectively. The arthritis onset and disease time-course are usually similar within the animals of an immunized model. However, there may be inter-animal variation in the severity level of the initial inflammatory symptoms. To provide reliable data, it is essential to perform preclinical trials in animals with similar joint involvement patterns (symmetry, number and types of joints involved) and similar synovial inflammation.

Synovial inflammation is the hallmark of RA and its severity varies with disease progression. While many well established clinical, laboratory, histologic, and imaging techniques exist to monitor synovial inflammation in humans, such techniques are less common in the preclinical setting. Various invasive and non-invasive techniques have been employed to detect and monitor inflammation at the onset and during the course of disease in RA animal models on a case by case basis. These techniques include (i) a clinical scoring system [8] which provides a subjective qualitative assessment of paw inflammation and it is usually coupled with (ii) thickness measurements of front and hind paws by means of a caliper [9]. It is also common to perform (iii) histopathology to assess pathological changes, including synovitis causing cellular infiltration, synovial hyperplasia, and bone and cartilage erosion [10]. To provide additional information about the disease progression, (iv) blood samples might be taken to determine total blood neutrophil and leukocyte counts [11] and measure serum pro-inflammatory cytokines such as integrin α2, IL-1β, IL-6, and TNF-α [12].

The above-mentioned techniques can be challenging due to their subjective nature, their invasiveness, and their extensive time consumption, especially in longitudinal experiments. For example, scoring of the number and degree of swollen and tender joints is investigator dependent, which makes the determination of disease status and progression challenging. Moreover, as the models involve chronic pain (level of chronicity depends upon the model), it is crucial to minimize invasive techniques in order to reduce animals suffering.

### Infrared thermal imaging (IRT): non-invasive imaging method for the screening of inducible arthritis models

Conventional radiography, ultrasonography, magnetic resonance imaging (MRI), and scintigraphy are non-invasive imaging techniques which are routinely employed for clinical arthritis assessment. However, their application in preclinical investigations for the detection of inflamed rheumatoid joints and monitoring inflammation has been hampered by high purchase and operating costs and time-consuming procedures [13, 14].

Inflammatory conditions, such as arthritis, typically exhibit a relatively higher skin temperature above the inflamed joint compared to healthy joints. In RA, increased blood flow accompanies the inflammatory reaction as well as increased tissue catabolism, leading to a rapid elevation in local temperature. Infrared thermal imaging (IRT) systems can detect this temperature rise through heat radiation (a non-contact heat transfer compared to conduction and convection methods) at wavelengths between 760 nm to 1 mm. Such radiation is emitted by waves from any object and is called infrared radiation or “thermal radiation” [15].

In clinical practice, IRT has been investigated and employed as a straightforward and cost-effective alternative to conventional imaging modalities for assessment of arthritis [16, 17], whereas in preclinical research, particularly for small rodents like rats and mice, IRT has been used for remote welfare assessment including heart activity, respiration, motion, wound analysis [18–20], as well as ocular surface temperature [21].

In RA, increasing joint temperatures indicate the presence of inflammatory reactions within the examined tissues and determines the dynamics of this process [22]. In recent years, several preclinical studies have been conducted using IRT tools to measure joint temperatures as a non-invasive indicator for the evaluation of RA models and/or to assess the efficacy of potential therapeutics [23–25]. However, surface temperature assessments are sensitive and may be influenced easily by several factors. The main ones include the housing situation (room temperature, humidity and air circulation), a body’s physiological condition, the handling procedure, and the camera positioning, which must be kept consistent to minimize surface temperature changes in mice. For example, capturing IR images of rodents while holding them in the hand or placing them inside their cage may cause variation in the rodents’ body temperature due to surface contact and environment dependent heat transfer. Failure to fix the mentioned factors can lead to inaccurate results.

In this study, we refined the IRT procedure by employing a fast, simple, and contactless setup using a digital infrared (IR) camera combined with a standardized image processing method for measuring surface body temperatures of mice. Also, we introduced a simplified temperature index as a non-invasive indicator of disease severity and a measure of the degree of inflammation [26]. The CIA rodent model is the most widespread immune-mediated RA model to study therapeutic efficacy due to its simplicity, rapid disease onset, and single disease pathology [27]. However, high inter-animal variability regarding the model’s onset, progression, and disease severity remains a big challenge [28, 29]. In this study, we examine non-invasively the onset of arthritis by means of IRT and select animals with similar arthritis severity for drug efficacy study.

As a proof of concept, we use IRT to evaluate the effectiveness of a drug delivery system that improves the efficacy of the small therapeutic molecule methotrexate (MTX) in CIA mice. MTX is the disease-modifying drug most commonly used in RA and the first choice of treatment [30]. However, 80% to 90% of MTX is eliminated by the kidneys within 24 h after intravenous administration, resulting in its short half-life and low drug concentration in target tissues [31]. Higher doses cannot be administered as they cause severe side effects [32]. Nanoparticles (NP) have been used widely to improve the therapeutic efficacy of RA drugs by increasing drug half-life, improving the hydrophobic drug’s solubility and releasing the drug in a controlled manner within the target joints [33, 34]. In this report, the therapeutic efficacy of MTX loaded polymeric NP was compared to that of free MTX in vivo in CIA mice.

## Methods

Thermographic imaging is accomplished with a camera that converts IR radiation into a visual image that depicts temperature variations across an object or scene. In this study, thermal images were obtained using the FLIR ONE camera (FLIR Systems, Inc., Wilsonville, OR, USA) attached to an iPhone (model 6, Apple, Inc., USA). FLIR ONE contains two cameras, a Lepton™ thermal camera with thermal resolution of 80 × 60 pixels and a VGA visible light camera with visual resolution of 1440 × 1080 pixels that simultaneously captures a digital photo in addition to the infrared image and merges them together using the Multi-Spectral Dynamic Imaging (MSX) fusion mode to create thermal images with enhanced detail and resolution. The free FLIR Tools software version 5.13 was used for image visualization and analysis.

The performance of the FLIR ONE camera is evaluated through a series of calibration methods. Then, we describe the image acquisition and processing technique applied to our data. Finally, IRT is introduced as a standardized technique in pre-clinical research through a series of in vivo experiments using the CIA animal model.

### IR thermal imager: performance evaluation

The FLIR ONE camera has a scene range temperature of − 20 °C to 120 °C and detects temperature differences as small as 0.1 °C (to an accuracy of ± 2 °C or 2% according to the manufacturer’s documentation). To evaluate the camera’s performance prior to use, a calibration check was performed using boiling water, melting ice and a person’s tear duct with known temperature values. To quantify the camera’s accuracy in the temperature measurement range of 35–40 °C, a test was performed against black body reference sources (Fluke Scientific 4180, Fluke-Hart, USA) with known emissivity at various temperatures. The coefficient of determination (*R*^2^) was used to demonstrate the variability explained by the linear regression between the black body and the IR temperature measurements. Each temperature reading was repeated 3 times and presented as average ± SD. Table 1 shows a list of objects used in the experiment.

**Table 1:** Emissivity values used in this study

Object	Temperature values (°C)	Emissivity
Boiling water	100	0.98
Melting ice	0	0.98
Tear duct	35	0.98
Black body source	35 to 40	0.95

The camera was adjusted horizontally parallel to the ground facing the boiling water and melting ice from a 20-cm distance. To image the tear duct and black body sources, the distance and subsequently camera’s field-of-view (FOV) were kept constant (~ 20 cm) while focusing on the object and minimizing the surrounding environment.

### IR image acquisition

**Fig. 1:**
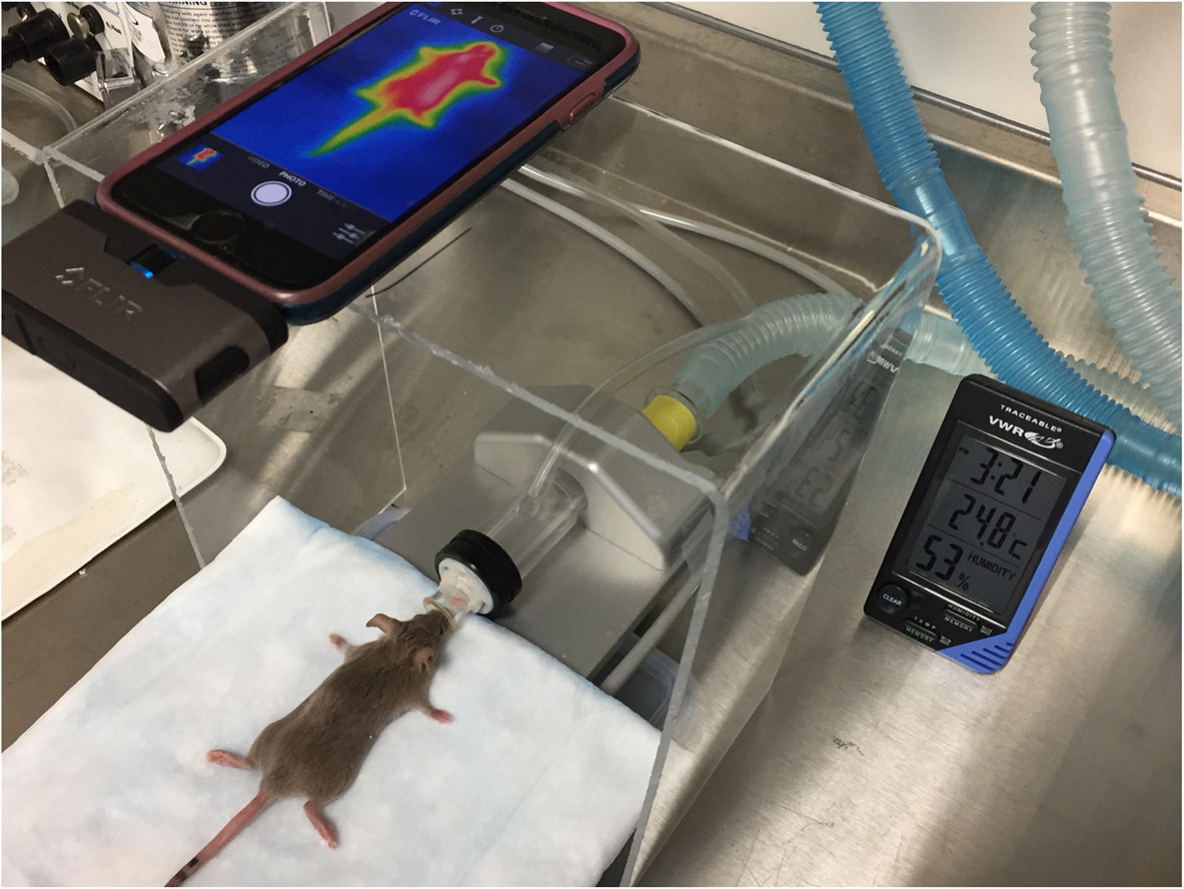
General set-up of the IR thermographic imaging station. A FLIR ONE camera attached to a smartphone illustrating field-of-view (FOV) for a representative anesthetized CIA mouse placed in prone position with extended front and hind legs away from the body

All experiments were performed at the same work bench, in the same room where the mice were kept throughout the whole study. The room was without windows, reduced airflow and equipped with fluorescent light to prevent IR radiation from outside and approximately at the same time (3:00 p.m.). To minimize infrared source interferences, the temperature, humidity, and air circulation of the imaging environment were controlled during the whole experiment. The measurement conditions were as follows: air humidity 50–55%, air and ambient temperature 24 ± 1 °C. All mice were handled and treated in the same way. Mice underwent chamber induction with 5% isoflurane delivered in oxygen at a flow rate of 2 L/min. Once the mice no longer responded to an interdigital pinch, they were transferred to a temperature-controlled metal plate (34 ± 1 °C) covered with a plain, non-reflective pad (multi-layer underpad with white top) to avoid direct contact between the mouse body and the metal plate and cover reflective surfaces in the background and vicinity of the animal. To eliminate the source of variability in the image capturing process, the smartphone mounted camera was placed on a stable surface above the imaging object to keep camera angles and object-camera distance constant at 20 cm (see Fig. 1). Mice were placed in prone position (connected to a nose cone under light anesthesia; 1.5% isoflurane) perpendicular to the axis of thermal scanning, with the front and hind legs extending away from the body showing the standard box size of 10 × 20 cm^2^.

### IR image processing

IR cameras record thermal information and display it in the form of a color map (color palette). Various color palettes are available which assign the temperature readouts values to a specific color to enable the analysis of a thermal image visually. Color assignment can be conducted both in the camera as well as the PC processing software. The captured thermographic images were analyzed by FLIR Tools software. We selected the “Iron” palette that runs from black representing coldest areas, through blue and magenta for slightly warmer areas, red, orange, and yellow for mid-range of temperatures, till turning to white for the hottest parts.

**Fig. 2:**
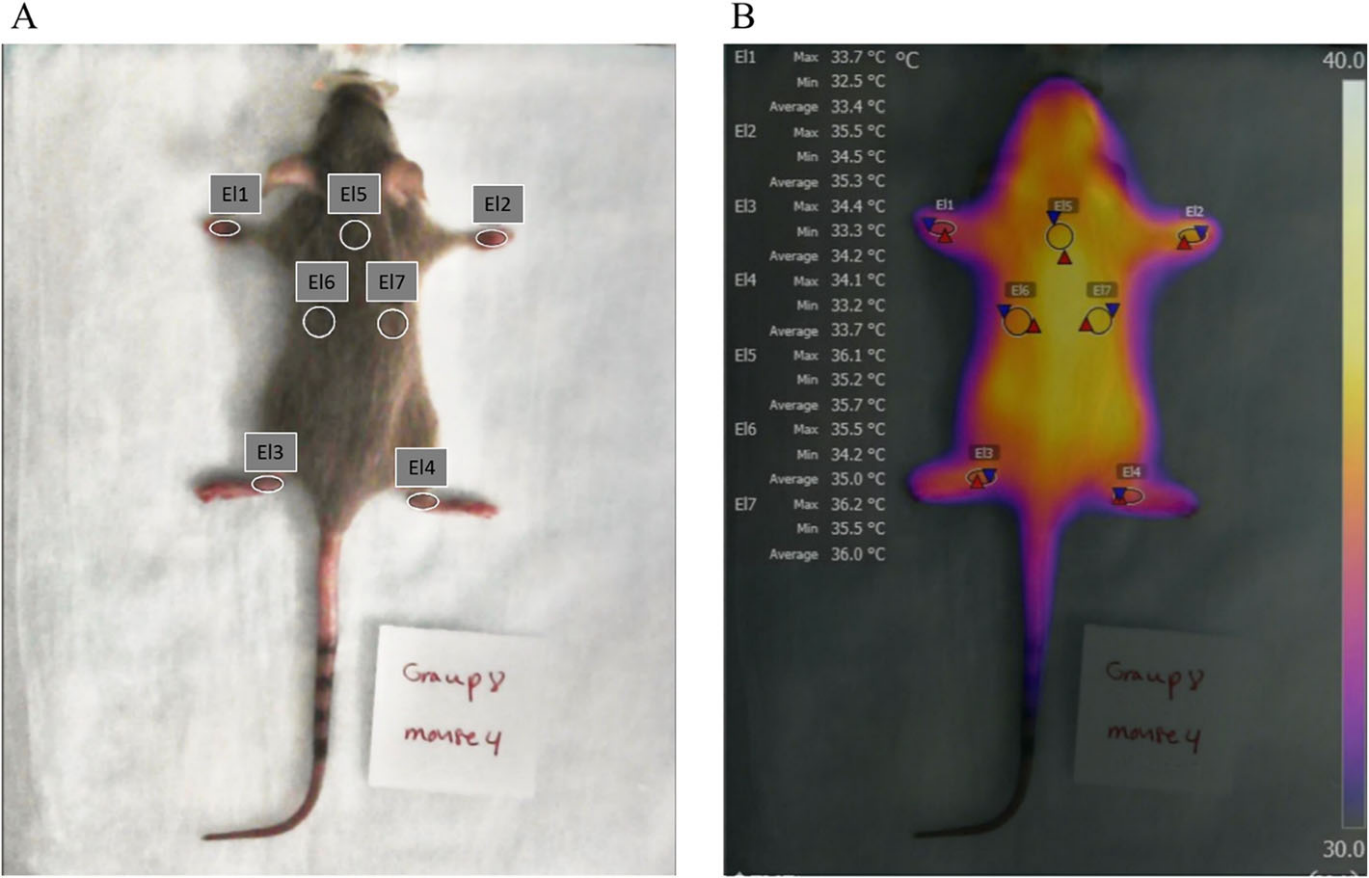
Images of the developed methodology. **a** The 7 different regions of interest (ROI) used for our experiments; 7 ellipsoids (El1-El7) on wrist joints of the front paws, ankle joints of the hind paws, and upper back. **b** An example of a thermal image loaded into the FLIR tool software. For each of the 7 ellipsoids (El1-El7), the minimum, maximum, and average temperature is given

The scale next to Fig. 2b shows the colors used and the temperature range covered. To obtain reproducible infrared temperatures, thermograms were adjusted for parameters of emissivity, reflected temperature, camera-object distance, atmospheric temperature, and humidity. Emissivity is a factor that corrects material-dependent differences between measured radiation and calculated IR temperature. In this study, we used emissivity values of 0.98 and 0.94 for the skin and fur of a mouse, respectively [35].

ROIs were defined and used to extract temperature parameters (mean temperature and standard deviation) to calculate the temperature index. Elliptical ROIs were placed over the front and hind paws and three positions on the mouse back (reference ROIs). ROIs were sized identically for the entire study. The mean temperatures (T) in each ROI (front left: FL; front right: FR; hind left: HL; and hind right: HR) were normalized to the average reference temperatures on the backs (*T*_back_) of each mouse to calculate the temperature index (TI) of an individual paw:

1 $$ {\mathrm{TI}}_{\mathrm{FL}}=\frac{T_{\mathrm{FL}}}{T_{\mathrm{back}}};{TI}_{FR}=\frac{T_{\mathrm{FR}}}{T_{\mathrm{back}}};{TI}_{\mathrm{HL}}=\frac{T_{\mathrm{HL}}}{T_{\mathrm{back}}};\mathrm{and}\ {TI}_{\mathrm{HR}}=\frac{T_{\mathrm{HR}}}{T_{\mathrm{back}}} $$

In addition, the total temperature index (TI_total_) for each mouse at each time point was calculated as the sum of wrist joint TIs of the front paws and the ankle joint TIs of the hind paws:

2 $$ {\mathrm{TI}}_{\mathrm{total}}={\mathrm{TI}}_{\mathrm{FL}}+{\mathrm{TI}}_{\mathrm{FR}}+{\mathrm{TI}}_{\mathrm{HL}}+{\mathrm{TI}}_{\mathrm{HR}} $$

### CIA induction

Female 7-week old DBA1/J mice weighing 18–20 g were purchased from Charles River, Canada, and acclimatized for 7 days prior to the start of the experiments. Four mice per cage were housed and maintained in a temperature-controlled (24 ± 1 °C) room with light cycles of 12 h (7:00 AM–7:00 PM) and 30 to 50% humidity. Applying refinement to the use of animals in arthritis research, we provided breeder diet, food and water, soft diet on the cage floor, and soft bedding during the whole study [36].

Arthritis induction was done by intradermal injection of an emulsion consisting of bovine type II collagen in complete Freund’s adjuvant (CFA), followed 21 days later by a booster immunization with type II collagen emulsified in incomplete Freund’s adjuvant (IFA). On day 25, to synchronize onset of arthritis, 2 mg/kg lipopolysaccharides (LPS) from *Escherichia coli* O111:B4 (Sigma) in 100 μL saline was injected intraperitoneally to synergistically trigger and enhance arthritis [37].

### IRT: a standardized framework to validate animal models of RA used in therapeutic studies

Arthritis onset and disease severity of CIA was investigated in the wrist joints of the front paws and the ankle joints of the hind paws of 14 female DBA1/J mice through paw thickness measurement, clinical arthritis scoring, and IRT. A control group of healthy mice received a sterile saline injection on the same day as the CIA group (*n* = 4). Prior to immunization, total paw thickness, clinical arthritis scores, and temperature index parameters were measured to obtain mean baseline values and continued weekly throughout the progression of disease up to 4 weeks.

For paw thickness measurements and clinical arthritis scoring, CIA mice were anesthetized with isoflurane (5% induction, 1.5–2% maintenance) in O_2_. Joints were measured with a digital caliper and noted in mm at each time point. The sum of the paw thicknesses in mm was also calculated as total paw thickness for each mouse. The clinical severity of arthritis in the paws was scored on a scale ranging from 0 to 4 based on a physical examination and visual inspection. The score was 0, normal; 1, mild redness of ankle or tarsal joints; 2, mild-moderate redness and swelling extending from ankle to the tarsals; 3, moderate redness and swelling from ankle to metatarsal joints; and 4, severe redness and swelling encompassing the ankle, foot, and digits. Each paw was evaluated and scored individually to calculate the total clinical score (CS) which is defined as the sum of the scores for each paw with a maximum possible score of 16 points.

Utility of IRT in the CIA model was evaluated as a quantitative tool to assess arthritis onset and identify optimal animals with the least inter-animal variability (disease onset, progression and severity parameters) leading to better study designs for efficacy assessment in drug development. IR images of individual mice were captured under 1.5–2% maintenance isoflurane anesthesia. Using FLIR Tool Software, IR images were corrected for parameters including emissivity, reflective and atmospheric temperature, reflective humidity, and camera-object distance. Elliptical ROIs shown in Fig. 2 were then drawn to delineate wrists and ankles and three ROIs on the back of the mouse. The average temperature of all pixels within each ROI in each image was calculated and recorded automatically and the temperature index calculated as described above. The TI of wrist joint of the front paws (TI_FL_ and TI_FR_) and the ankle joints of the hind paws (TI_HL_ and TI_HR_), with maximum score of 1, were evaluated individually to assess the presence and severity of joint inflammation at different time points. The TI_total_, i.e., the sum of the TIs for all paws, was calculated with the maximum possible score of 4.

Severity classification across different techniques was derived based on our experimental results to explain absent (TI 0.80–0.85, THK 1.5–2 mm, and CS 0), mild (TI 0.85–0.9, THK 2–2.5 mm, and CS 1), moderate (TI 0.9–0.95, THK 2.5–3 mm, and CS 2, 3), or severe (TI 0.95–1, THK 3–3.5 mm, and CS 4) level of arthritis. Animals classified on day 28 as having moderate to severe arthritis by a cut-off value of TI 0.9, THK 2.5, and CS: 2 determined by IR imaging, joint thickness measurement, and clinical arthritis scoring, respectively. To minimize inter-animal variability among CIA mice entering the therapeutic trial, mice scored with TI below a threshold of 0.9 (meaning mild or absent inflammation) in wrist joints of the front paws and/or the ankle joints of the hind paws were excluded from the study.

### Therapeutic study

CIA mice (*n* = 12) with fully developed RA (wrist joints of the front paws and the ankle joints of the hind paws scored: 0.9 ≤ TI ≤ 1) were randomly divided into 3 groups (*n* = 4) and received treatment weekly up to 4 weeks (Table 2). CIA mice were treated intravenously with either free MTX (3 mg kg^−1^), NP-MTX (3 mg kg^−1^ formulated MTX) or saline (positive control). Healthy mice (*n* = 4) used as a negative control received weekly saline injections.

**Table 2:** Grouping of animals and dose administration chart

Group	Type of animal	*N*	Treatment	Dosing frequency	MTX dose (mg/kg)
1	CIA	4	NP-MTX	Weekly	3
2	CIA	4	MTX	Weekly	3
3	Control (+)	4	Saline	Weekly	–
4	Control (−)	4	Saline	Weekly	–

*CIA* collagen-induced arthritis, *N* number of animals per study group, *MTX* methotrexate, *NP-MTX* nanoparticulate methotrexate

Disease progression was examined before treatment (baseline) and every 3 (or 4) days during treatment independently with caliper measurements, clinical arthritis scoring, and IR imaging. After 4 weeks of treatment, animals were euthanized by CO_2_ inhalation, followed by cervical dislocation. Hind paws of mice were harvested, fixed, embedded in paraffin, and cut into 4-μm thick sagittal sections. For histology assessment of ankles, slides stained with hematoxylin and eosin (H&E) were scored by a certified Pathologist at HistoWiz Inc. (histowiz.com) on a scale of 0–3, with respect to degree of inflammation, synovial hyperplasia, cartilage damage, and bone erosion.

### Statistics

Statistical analyses were performed using advanced statistical tests in OriginLab 9.6 Pro software. The Shapiro-Wilk test was applied to evaluate the normality of data. For continuous normally distributed data, the Student *t* test was used, and to explore differences between ordinal variables, the Mann-Whitney test. To evaluate correlations between continuous-continuous variables, Pearson’s correlation was used, and between continuous-ordinal variables Kendall correlation. A *P* value below 0.05 was considered statistically significant.

## Results

### IR thermal imager: performance evaluation

**Fig. 3:**
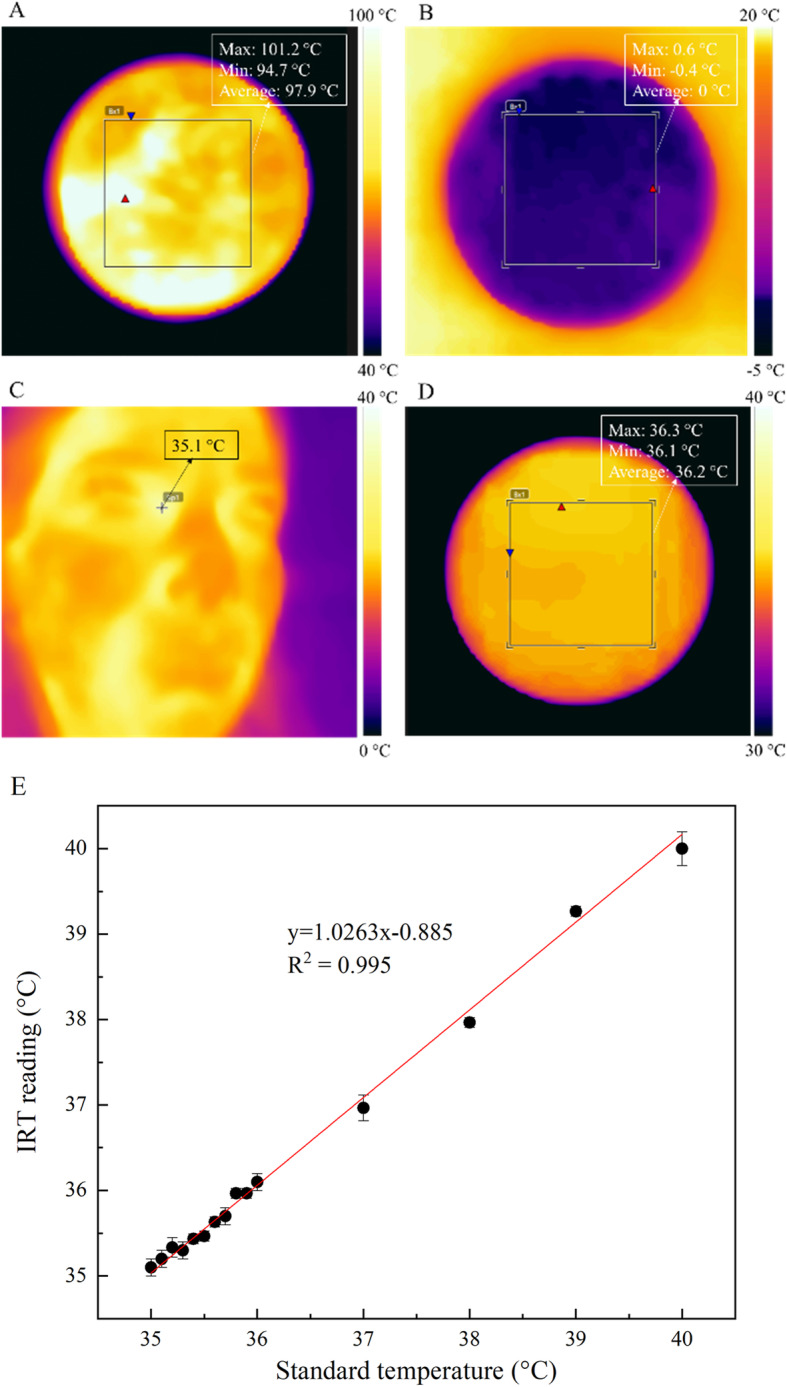
IR camera functionality and accuracy check. Temperature maps for the IR camera while looking at **a** boiling water, **b** melting ice, **c** tear duct, and **d** black body with a temperature of 36 °C. The ROIs were defined as square boxes to measure the minimum, maximum, and average temperature for boiling water, melting ice, and blackbody while for the tear duct measurement, the spot measure tool was focused on tear duct. **e** The plot of the IRT reading vs. standard temperature values for given black body temperatures showed linear relationship between IRT measurements and reference black body temperature within 35–40 °C temperature range. Each point represents the average of 3 separate readings and the linear regression line is shown in red

To validate the performance of the IR thermal imager prior to the animal study, a common functionality check was implemented, and the captured temperature profiles analyzed. The IRT measurements of melting ice (Fig. 3b) and tear duct (Fig. 3c) showed an agreement within ± 0.1 °C of their known temperature values. However, the IR camera performed relatively poor at detecting the absolute temperature value of boiling water at 100 °C underestimating the target temperature by ~ 2.4 °C (Fig. 3a). For this reason, we further verified the performance and quantified the accuracy of the IR camera with a number of black body measurements (Fig. 3d) at known temperatures, emissivity, and camera to target distances. A summary of the IRT readings vs. standard temperatures is shown in Fig. 3e. The linearity between measured temperature values and the actual temperature of the black body showed a coefficient of determination *R*^2^ = 0.995. The accuracy within the 35–40 °C range was ± 0.2 °C, proving that the IR camera had good accuracy in the temperature range of mouse bodies [26].

### IRT: a standardized framework to validate animal models of RA used in therapeutic studies

**Fig. 4:**
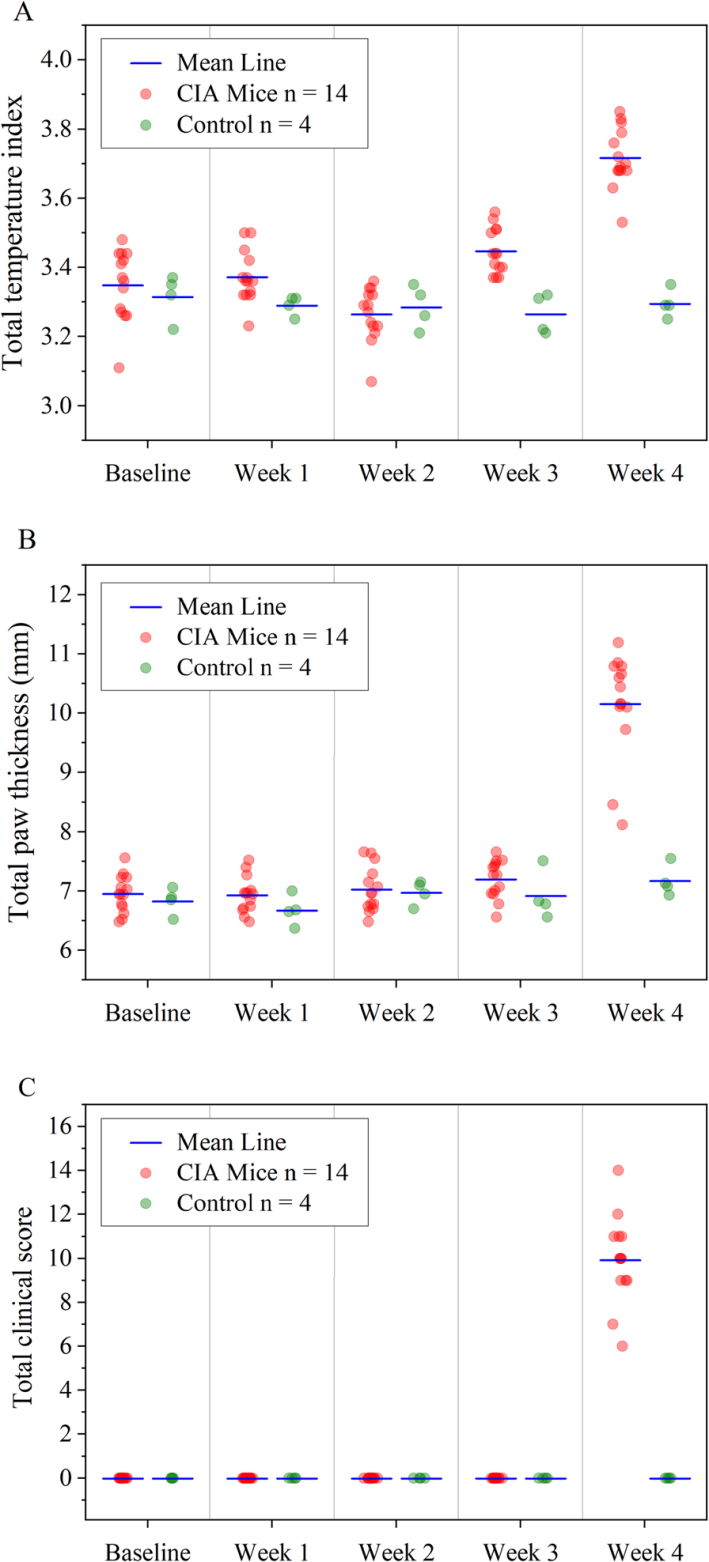
Analysis of the progression, onset, and severity of arthritis in CIA mouse model. RA severity was monitored based on **a** temperature assessment, **b** paw thickness, and **c** the clinical arthritis score during 4 weeks after initial immunization. All results are presented as mean and each symbol represents one individual mouse (CIA, *n* = 14, control, *n* = 4) per time point

To validate IRT as a standardized non-invasive framework to monitor RA animal models, we quantified the disease progression, arthritis onset, and severity in healthy non-immunized (*n* = 4) and CIA immunized (*n* = 14) mice by measuring the temperature index and compared it to the current gold standards, paw thickness, and clinical scores. The interval plots (Fig. 4) show the variation and mean values of the measured parameters over the 4 weeks of RA induction. At baseline, the IR imaging parameter TI_total_ (± SD) was 3.37 ± 0.12 and 3.31 ± 0.06, for CIA and control mice, respectively (Fig. 4a). Similarly, no significant differences between CIA and control mice were observed up to 3 weeks (*P* > 0.05). However, the TI_total_ increased significantly to a mean value of 3.71 (range = 3.53–3.85) on day 28 (*P* < 0.05; *t* test two-tailed with independent means), while the TI_total_ of control mice stayed at baseline. While most of the positive control mice already show a temperature increase at the time of the secondary immunization, they further progress to full blown inflammation 48 h after *LPS* injection in the CIA mouse model [38]. A more detailed view of the TI_total_ development in individual mice is described in the discussion. Similarly, the clinical arthritis score of front and hind paws in CIA mice and saline injected mice for the first 3 weeks (Fig. 4c) was not significantly different (*P* > 0.05). Forty-eight hours after LPS injection on day 28, the total clinical score increased significantly to 10 (range 8–14; *P* < 0.05; Mann-Whitney test). Lastly, the severity of paw swelling was determined by measuring the thickness of wrists and ankles at baseline and continued weekly up to 4 weeks. In week 4, 48 h after LPS injection, the disease progressed in CIA mice and the mean total thickness of the wrists and ankles (Fig. 4b) significantly increased to 10.15 ± 0.88 mm vs. 7.17 ± 0.26 mm in control mice (*P* < 0.05; *t* test two-tailed with independent means).

**Fig. 5:**
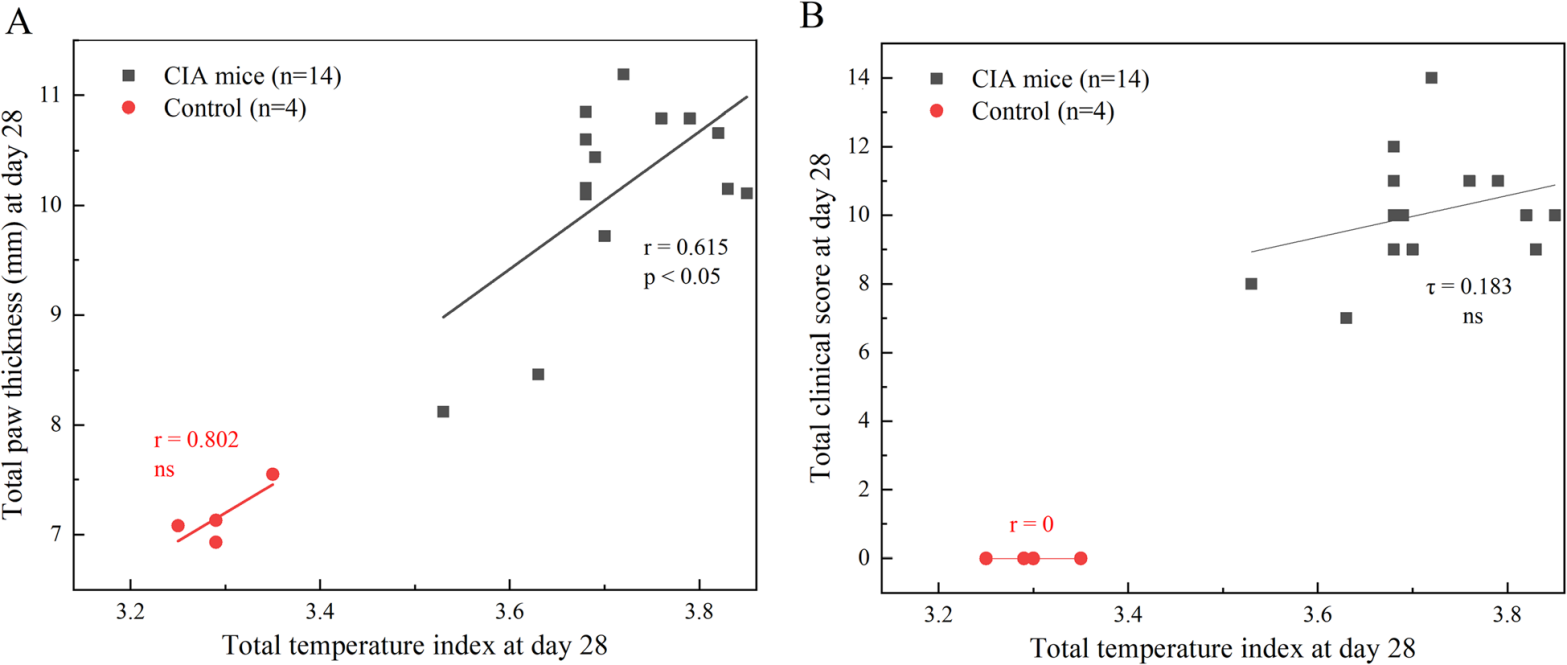
Scatter plot showing the correlation between **a** the total temperature index and total paw thickness and **b** the total temperature index and total clinical score, in CIA mice (black squares). Control mice are shown in hollow red circles. Linear regression lines were plotted in black for CIA and red for control mice. *r* = Pearson correlation coefficient, *τ* = Kendall’s correlation efficient, ns = non-significant

We estimated the correlation between IRT, paw thickness measurement, and clinical scoring to show the potential of IRT as an alternative non-invasive technique with less inter-reader variability in measuring disease progression in longitudinal preclinical studies of the RA mouse model (Fig. 5). The total temperature index shows a relatively positive correlation (Pearson’s *r* = 0.615, *P* < 0.05) with total paw thickness measurements on day 28, while Kendall’s co-efficient of *τ* = 0.1830 and *P = 0.394* indicates no significant relationship between total temperature index and total clinical score in CIA. These results suggest that the temperature index mirrors the standard inflammation parameter of paw thickness better in CIA mice since both variables are continuous. The absence of correlation between total temperature index and total clinical score in CIA could be explained as the nature of variables (continuous and ordinal). The positive correlation between the total temperature index and total paw thickness (*r* = 0.802) in control mice was not significantly different that can be described due to small sample size (*n* = 4).

**Fig. 6:**
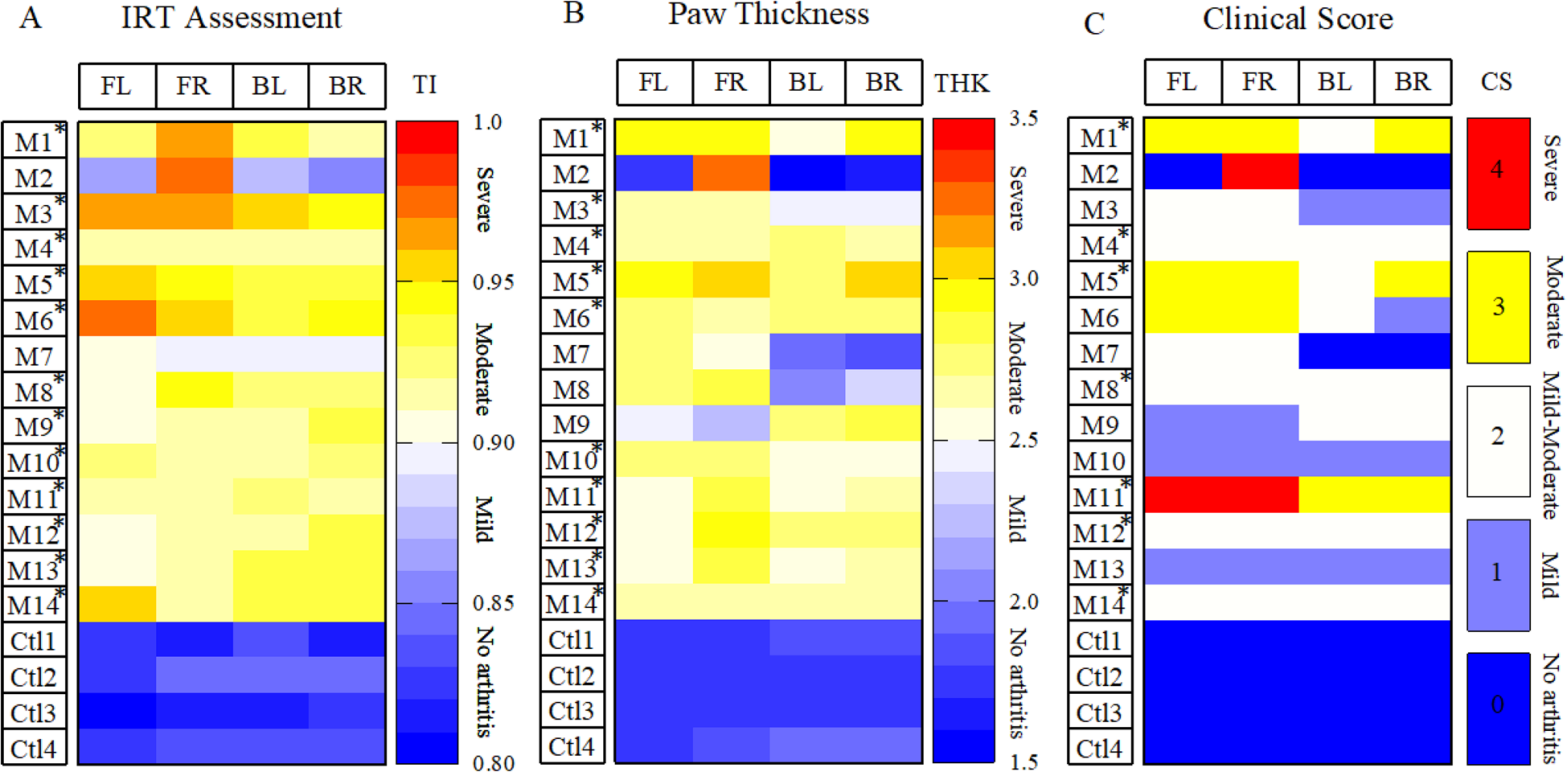
Heatmaps showing severity arthritis criteria. Disease severity quantification through **a** joint temperature assessment, **b** paw thickness measurement (mm), and **c** clinical score analysis of individual paws in 14 CIA (M1 to M14) and 4 control (Ctl1 to Ctl4) mice on day 28. Colors represent the score assigned to the severity of inflammation where darker blue represents absence of arthritis, and white, yellow, and red indicate mild to severe form of arthritis in each category. Asterisk corresponds to mice identified as “moderate to severe” based on each assessment method. FL, front left; FR, front right; HL, hind left; HR, hind right; TI, temperature index; THK, thickness; CS, clinical score; M, mouse; Ctl, control

Classification of arthritis severity for each assessment technique was based on our own experimental results. According to Fig. 6, the temperature assessment, paw thickness measurement, and clinical scoring identified 86% (12 of 14) mice showing mild to severe signs of arthritis (TI 0.85–1, THK 2–3.5 mm, and CS 1–4). For monitoring anti-arthritic drug efficacy, animals that developed moderate to severe disease were chosen. Mice were defined as having moderate to severe arthritis when all 4 joints (wrist joints of the front paws and the ankle joints of the hind paws) develop arthritis with TI ≥ 0.9 (or THK ≥ 2.5 mm or CS ≥ 2).

Based on IRT (Fig. 6a), 12 of 14 mice had a moderate to severe level of disease, while this number was lower based on paw thickness assessment (9 of 14 mice) and clinical scoring (7 of 14 mice). Based on IRT assessment, mouse 2 and mouse 7 (M2 and M7) with no or mild symptoms scored a TI below 0.9 for 3 out of 4 paws. The absence of inflammation within those paws was confirmed with paw thickness measurements and clinical scoring (Fig. 6b, c), which led us to exclude these animals from the therapeutic study.

### Therapeutic study

**Fig. 7:**
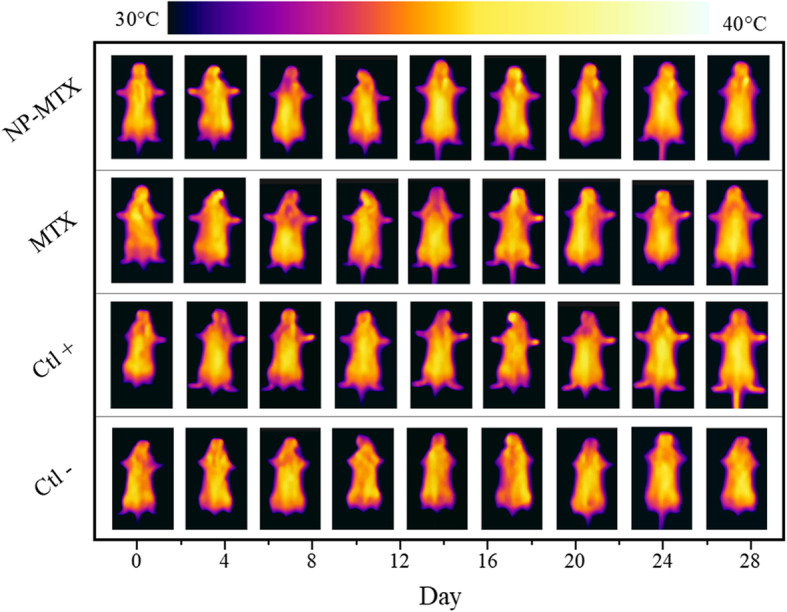
Comparison of the therapeutic response in CIA mice by IR imaging. One representative mouse per treatment group is shown over time, reflecting temperature changes

CIA mice with a TI score ≥ 0.9 (wrist joints of the front paws and the ankle joints of the hind paws) were equally distributed into 3 different treatment groups. The temperature measurements of front and hind paws in CIA and control mice (described as TI_total_) over 28 days show that the treatment progression in mice administered with NP-MTX proceeded better compared to treatment with free MTX (Fig. 7). The highest TI_total_ was observed in the positive control mice (Ctl+), whereas the lowest TI_total_ occurred in the healthy control mice (Ctl−).

**Fig. 8:**
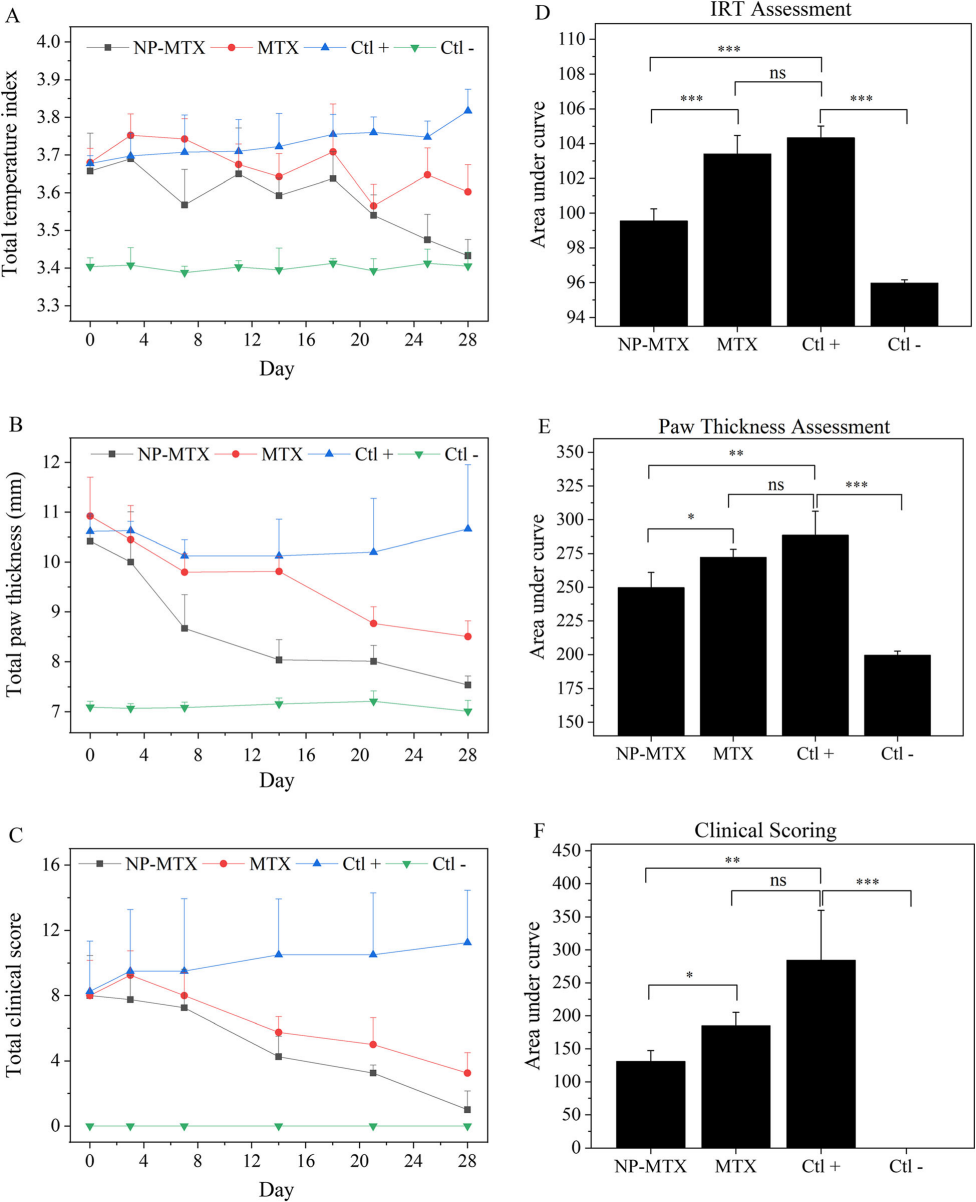
Efficiency of NP-MTX in the CIA model. **a** Temperature assessment, **b** paw thickness, and **c** clinical score in CIA-mice treated with either NP-MTX, MTX, or vehicle. Area under the curve of **d** IRT assessment, **e** paw thickness, and **f** clinical score measurements during the entire treatment period. The line graphs and columns show mean values +SD; *n* = 4. The *P* values between the means are as follows: ***P < 0.001; **P < 0.01; *P < 0.05; ns, non-significant

The positive control mice exhibited a progressive increase in the TI_total_ accompanied by increased thickness of paws and higher clinical score compared to healthy and treated mice over time (Fig. 8). The treatment with NP-MTX led to a significant reduction of cumulative TI_total_ (*P* < 0.001), total paw thickness (*P* < 0.05), and total clinical score (*P* < 0.05) compared to mice treated with MTX (Fig. 8d–f) or CIA mice treated with vehicle (Ctl+). No statistically significant changes were found between CIA mice treated with weekly MTX or vehicle (*P* values = 0.195, 0.067, and 0.066 obtained from IRT, paw thickness assessment, and clinical arthritis scoring, respectively), although MTX tended to invoke a stronger treatment response than the vehicle.

**Fig. 9:**
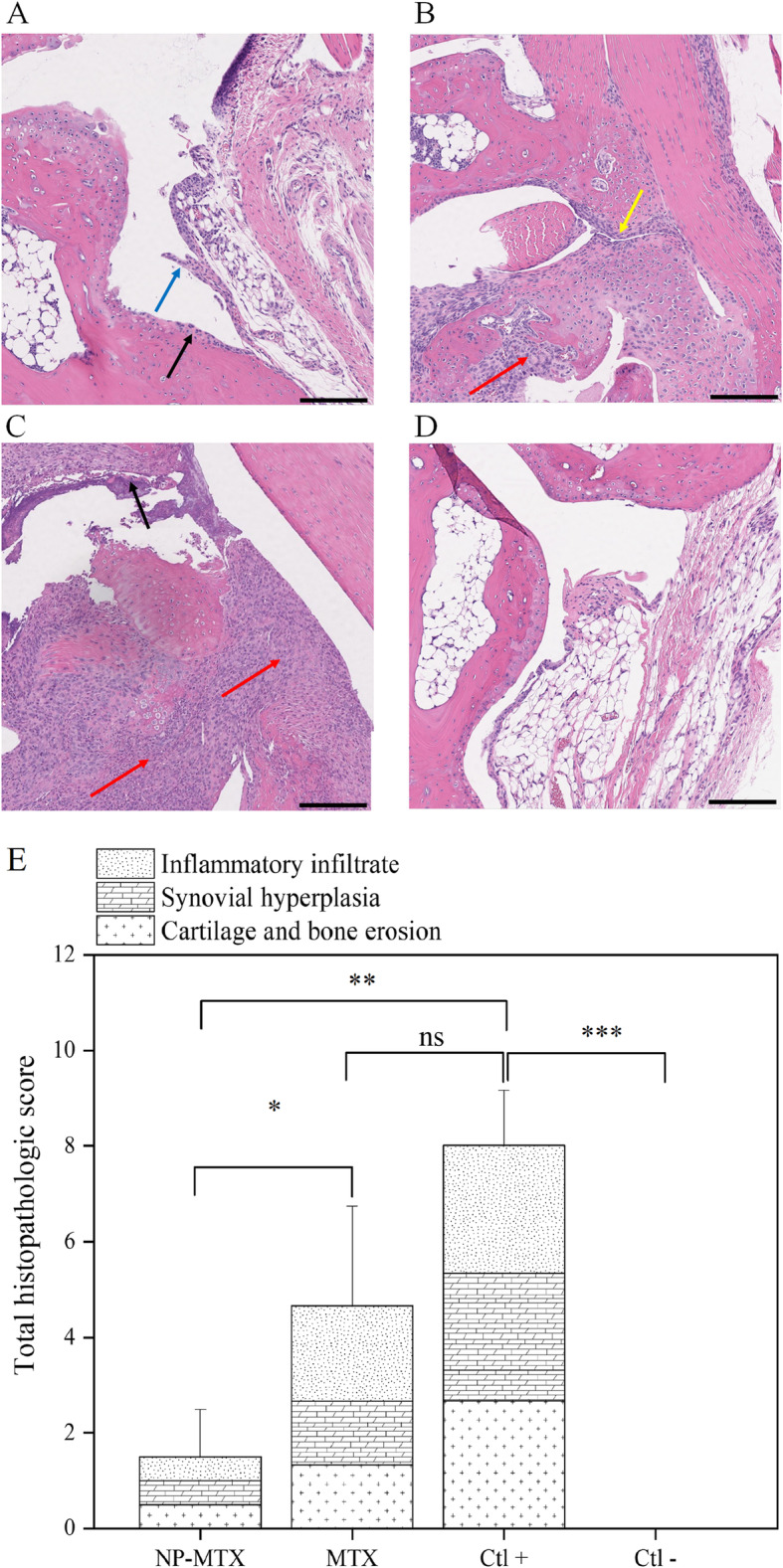
Representative optical microscopy sections of H&E stained ankle joints. CIA mice treated with **a** NP-MTX, **b** MTX, and **c** vehicle. **d** Healthy mice treated with vehicle are used as negative control. **e** A histopathologic score was determined based on arthritis severity for all treatment groups and the controls and graphed as mean + SD; *n* = 4. The *Y*-axis score is cumulative, where higher scores equal an increase in severity. Red arrow, inflammatory cell infiltration; blue arrow, synovial hyperplasia; black arrow, cartilage erosion; and yellow arrow, synovial proliferation. The *P* values between the means are as follows: ***P < 0.0005; **P < 0.001; *P < 0.05; ns, non-significant. Scale bars = 100 μm

In the context of monitoring disease activity, histological examination provides additional information pertaining to cartilage and bone damage. In line with other assessment techniques, histological scoring of the ankle joints revealed a similar reduction in disease activity in CIA mice treated with NP-MTX versus MTX. Representative images from the tissue histopathology analyses of the ankle joints isolated at the end of the in vivo experiment are shown in Fig. 9. Compared to healthy control animals (Fig. 9d), histological analysis revealed inflammatory cell infiltration, synovial hyperplasia, and bone destruction in all treatment groups (Fig. 9a–c). Mice treated with NP-MTX displayed the most significant reduction in total histopathologic score compared to CIA mice treated with MTX (*P* < 0.05) as well as CIA mice treated with vehicle (*P* < 0.001). No significant changes were found between CIA mice treated with weekly MTX and vehicle (*P* > 0.05).

## Discussion

Rodent models have been used widely in the preclinical assessment of novel RA treatment strategies. The most used CIA model in DBA/1 J mice shares both immunological and pathological features with human RA including symmetric joint involvement, synovitis, and cartilage and bone erosions [39]. To detect arthritis onset, and monitor disease progression in these models, clinical scoring and paw thickness measurement have been widely employed despite having some limitations [9]. Clinical scoring is a semi-quantitative method where the assessment by the researcher may be subjective, therefore being exposed to biases and degree of agreement varying among raters [40]. While paw thickness assessments are quantitative, arthritic animals are in chronic pain and may experience pain and distress during handling and joint measurements under caliper compression [36, 41, 42]. The recommendation to perform caliper measurements as gentle as possible might thus result in inaccurate readings.

To overcome limitations from both conventional methods, we evaluated IRT to determine RA disease progression and treatment efficacy. IRT is a non-invasive imaging-based technique where the raw data can be stored safely as temperature profile images and analyzed at any time by different people repeatedly with minimal inter-reader variation [43].

The difference of our work with previous attempts in using IRT consists of the development of a standardized framework to perform contactless and reproducible measurements that detect temperature changes in RA animal models. To minimize camera displacement, we fabricated and used a simple stand with fixed camera position at a defined distance that covers the entire animal body with a single scan at each timepoint. The distinct and repeatable positioning of the animals assured consistent and reproducible results for preclinical longitudinal studies. We also validated the IR camera performance and accuracy through temperature standard measurements, including a set of black body measurements within a mouse’s body temperature range. The linear relationship between the actual and measured temperatures proved the reliability and accuracy (± 0.2 °C) of the IR camera. We further introduced a “total temperature index” as a simple indicator of overall temperature changes within inflamed paws to be used in longitudinal studies.

The usefulness of IRT in preclinical RA drug efficacy studies was validated in an in vivo treatment study compared to the gold standards (paw thickness measurement and clinical arthritis scoring). Mice with similar degrees of inflammation were chosen by means of IRT and their arthritic onset, disease progression, and severity quantified and compared to healthy control and CIA immunized mice. Our newly introduced temperature index correlates very well with the conventional technique of paw thickness measurements. We thus conclude that IRT is a highly valid technique for the choice and evaluation of RA animal models and should replace the more invasive paw thickness measurement method. A combination of IRT and clinical scoring might also be helpful and facilitate a more comprehensive judgment.

To obtain more accurate results in a preclinical RA drug efficacy study, it is crucial to minimize variability in the test animals and use a robust animal model. Based on our data, IRT is an excellent tool for choosing animals at a proper stage of disease. A common scenario is that animals chosen for a therapeutic trial only show mild or no disease symptoms. The researcher has to choose whether to (i) exclude the animal from the study which causes loss of statistical power and a biased conclusion or to (ii) use an imperfect animal model that leads to an inaccurate estimation of the treatment efficacy [44]. Similarly, it might happen that animals with clinically no apparent symptoms are excluded from studies due to a lack of available techniques able to detect subclinical symptoms. This issue could be addressed by increasing the number of animals to improve statistical power [45]; however, increasing the number of animals to compensate for sub-optimal inclusion assessment does not comply to the ethical guidelines and it can be expensive and time-consuming. Therefore, it is pivotal to enhance the pre-screening process in order to identify the most relevant animals at the proper disease stage.

**Fig. 10:**
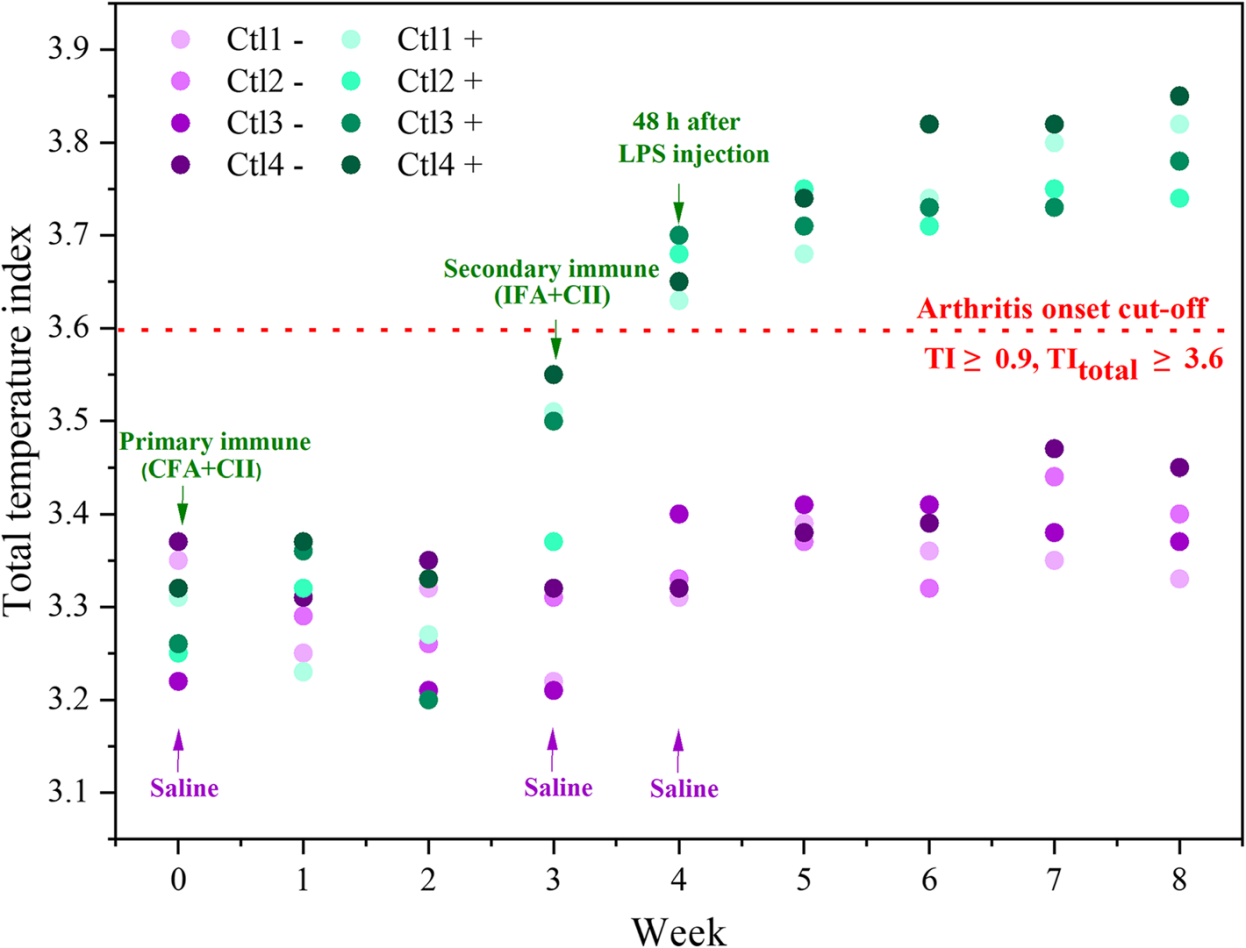
Total temperature index (TI_total_) changes of individual control mice over time. Individual mice are depicted by different colors. Negative control mice (Ctl−, *n* = 4) shown in purple circles only received saline and positive control mice (Ctl+, *n* = 4) shown in green circles received immunization

For example, in the CIA model, subclinical synovitis and early erosions are clinically non-apparent and joints might look normal despite the presence of activated macrophages and cellular infiltration [46]. Thus, clinical scoring at the early stage of arthritis onset often underestimates the number of joints with active arthritis [47]. Thermal imaging can potentially detect temperature changes due to increased vascularity in synovial inflammation even before the presence of visually visible clinical signs [48]. In our proof-of-concept study, IRT assessment allowed to detect subclinical changes at earlier stages in CIA induced mice with moderate to severe arthritis compared to the conventional techniques. Following single positive control mice by IRT points not only to a trend of earlier detection of inflammation, but also to an indication of more severe inflammation in animals that react earlier (Fig. 10). Predicting the severity of joint inflammation might be very valuable in this animal model and increase the statistical power of the studies, as animals that do not develop arthritis and thus dilute the findings can be completely excluded. However, the group size of our study and the IRT sampling frequency was not sufficient to obtain significant correlations between early onset and late disease severity, and would have to be investigated in a future study.

That fewer animals with moderate to severe arthritis were detected through clinical arthritis scoring and paw thickness measurements can be explained by the absence of clinical signs and structure-based changes, respectively, at the early onset of arthritis. By the end of the study, all three techniques agreed on an 86% arthritis incidence rate (including mild, moderate and severe types) [39]. Thus, IRT can be regarded as a non-invasive straight forward tool for screening CIA mice (and potentially rats) in translational research to minimize the inter-animal variations in terms of disease severity and involvement of inflamed paws.

To assess IRT’s ability to evaluate a RA drug’s efficacy, we tested it in parallel to clinical arthritis scoring and paw thickness measurement. Efficacy of methotrexate (MTX), which is still the gold-standard of anti-rheumatoid arthritis drugs, was investigated in comparison to a novel nanoparticulate methotrexate (NP-MTX). MTX’s therapeutic efficacy has been limited due to its low bioavailability, and previous studies have reported that nanoparticulate systems can enhance its pharmacological profile [49, 50]. All three measurement techniques employed proved that the same drug dose in the form of NP-MTX compared to free MTX was more effective and significantly (1) decreased the temperature over the inflamed joints’ skin surface, (2) reduced the increase in paw thickness, (3) improved the clinical signs of arthritis, and (4) reduced histologic evidence of inflammation and prevented bone erosion in the joints.

IRT is not only a superior method of evaluating and choosing animals with inflamed joints, but also an effective and unbiased tool to screen novel anti-inflammatory agents in a rapid, accurate, and highly reproducible manner in longitudinal preclinical studies. Overall, drug development in diseases with inflamed joints will be more reliable based on sound and precise physiological temperature measurements, which in turn will accelerate the translation of precilinical data into clinical studies.

## Conclusion

Modern inexpensive digital infrared (IR) cameras are useful and precise tools for evaluating disease activity (and arthritis severity) in pre-clinical and drug development studies working with the CIA mouse model. We present here a standardized setup, where this simple non-contact method can be used rapidly, reproducibly, and non-invasively to accurately quantify the degree of inflammation. A temperature index is introduced, which is able to describe the disease severity in a preclinical setting. Based on our in vivo data, we recommend IRT as the main technique for longitudinal preclinical RA drug efficacy studies, as it allows to reduce experimenter bias, as well as animal discomfort. The use of IRT in other inflammatory diseases might also benefit from this technique and should be further investigated.

## Data Availability

The datasets during and/or analyzed during the current study are available from the corresponding author on reasonable request.

## Abbreviations

AIA: Antigen-induced arthritis
CCAC: Canadian Council on Animal Care
CCM: Centre for Comparative Medicine
CFA: Complete Freund’s adjuvant
CIA: Collagen-induced arthritis
COMP: Cartilage oligomeric matrix protein
Ctl: Control
CS: Clinical score
El: Ellipsoid
FL: Front left
FOV: Field-of-view
FR: Front right
HL: Hind left
HR: Hind right
IFA: Incomplete Freund’s adjuvant
IL: Interleukin
IR: Infrared
IRT: Infrared thermal imaging
LPS: Lipopolysaccharides
M: Mouse
MRI: Magnetic resonance imaging
MSX: Multi-spectral dynamic imaging
MTX: Methotrexate
NP: Nanoparticle
NP-MTX: Nanoparticular methotrexate
*r*: Pearson’s correlation coefficient
*τ*: Kendall’s correlation efficient
RA: Rheumatoid arthritis
ROI: Region of interest
SD: Standard deviation
T: Temperature
THK: Thickness
TI: Temperature index
TNF: Tumor necrosis factor

## References

[1] Bioethics NCo (2005). The ethics of research involving animals: Nuffield Council on Bioethics.

[2] Hegen M, Keith JC, Collins M, Nickerson-Nutter CL (2008). Utility of animal models for identification of potential therapeutics for rheumatoid arthritis. Ann Rheum Dis.

[3] Howson P, Shepard N, Mitchell N (1986). The antigen induced arthritis model: the relevance of the method of induction to its use as a model of human disease. J Rheumatol.

[4] Brackertz D, Mitchell GF, Mackay IR (1977). Antigen-induced arthritis in mice. I. Induction of arthritis in various strains of mice. Arthritis Rheum.

[5] Hunneyball IM, Crossley MJ, Spowage M (1986). Antigen-induced arthritis in mice. Br J Clin Pract Suppl.

[6] Trentham DE, Townes AS, Kang AH (1977). Autoimmunity to type II collagen an experimental model of arthritis. J Exp Med.

[7] Carlsen S, Hansson AS, Olsson H, Heinegard D, Holmdahl R (1998). Cartilage oligomeric matrix protein (COMP)-induced arthritis in rats. Clin Exp Immunol.

[8] Mossiat C, Laroche D, Prati C, Pozzo T, Demougeot C, Marie C (2015). Association between arthritis score at the onset of the disease and long-term locomotor outcome in adjuvant-induced arthritis in rats. Arthritis Res Ther..

[9] Lim MA, Louie B, Ford D, Heath K, Cha P, Betts-Lacroix J (2017). Development of the digital arthritis index, a novel metric to measure disease parameters in a rat model of rheumatoid arthritis. Front Pharmacol.

[10] Krenn V, Morawietz L, Haupl T, Neidel J, Petersen I, Konig A (2002). Grading of chronic synovitis--a histopathological grading system for molecular and diagnostic pathology. Pathol Res Pract.

[11] Szekanecz Z, Halloran MM, Volin MV, Woods JM, Strieter RM, Kenneth Haines G (2000). Temporal expression of inflammatory cytokines and chemokines in rat adjuvant-induced arthritis. Arthritis Rheum.

[12] Gwon SY, Rhee KJ, Sung HJ (2018). Gene and protein expression profiles in a mouse model of collagen-induced arthritis. Int J Med Sci.

[13] Chamberland D, Jiang Y, Wang X (2010). Optical imaging: new tools for arthritis. Integr Biol (Camb).

[14] Sudol-Szopinska I, Jans L, Teh J (2017). Rheumatoid arthritis: what do MRI and ultrasound show. J Ultrason.

[15] Bernard V, Staffa E, Mornstein V, Bourek A (2013). Infrared camera assessment of skin surface temperature--effect of emissivity. Phys Med.

[16] Gizińska M, Rutkowski R, Szymczak-Bartz L, Romanowski W (2020). Straburzyńska-Lupa A.

[17] Lasanen R, Piippo-Savolainen E, Remes-Pakarinen T, Kroger L, Heikkila A, Julkunen P (2015). Thermal imaging in screening of joint inflammation and rheumatoid arthritis in children. Physiol Meas.

[18] Pereira C, Kunczik J, Bleich A, Haeger C, Kiessling F, Thum T (2019). Perspective review of optical imaging in welfare assessment in animal-based research. J Biomed Opt.

[19] Nääs IA, Garcia RG, Caldara RR (2014). Infrared thermal image for assessing animal health and welfare. J Anim Behav Biometeorol.

[20] Pereira CB, Kunczik J, Zieglowski L, Tolba R, Abdelrahman A, Zechner D (2018). Remote welfare monitoring of rodents using thermal imaging. Sensors..

[21] Vogel B, Wagner H, Gmoser J, Worner A, Loschberger A, Peters L (2016). Touch-free measurement of body temperature using close-up thermography of the ocular surface. MethodsX..

[22] Frize M, Adéa C, Payeur P, Di Primio G, Karsh J, Ogungbemile A (2011). Detection of rheumatoid arthritis using infrared imaging. Proceedings of SPIE - The International Society for Optical Engineering.

[23] Sanchez BM, Lesch M, Brammer D, Bove SE, Thiel M, Kilgore KS (2008). Use of a portable thermal imaging unit as a rapid, quantitative method of evaluating inflammation and experimental arthritis. J Pharmacol Toxicol Methods.

[24] Snekhalatha U, Anburajan M, Venkatraman B, Menaka M (2013). Evaluation of complete Freund’s adjuvant-induced arthritis in a Wistar rat model. Z Rheumatol.

[25] Calkosinski I, Dobrzynski M, Rosinczuk J, Dudek K, Chroszcz A, Fita K (2015). The use of infrared thermography as a rapid, quantitative, and noninvasive method for evaluation of inflammation response in different anatomical regions of rats. Biomed Res Int.

[26] Jasemian Y, Svendsen P, Deleuran B, Dagnaes-Hansen F (2011). Refinement of the collagen induced arthritis model in rats by infrared thermography. Brit J Medicine Medical Res.

[27] Bevaart L, Vervoordeldonk MJ, Tak PP (2010). Evaluation of therapeutic targets in animal models of arthritis: how does it relate to rheumatoid arthritis?. Arthritis Rheum.

[28] Asquith DL, Miller AM, McInnes IB, Liew FY (2009). Animal models of rheumatoid arthritis. Eur J Immunol.

[29] Caplazi P, Baca M, Barck K, Carano RA, DeVoss J, Lee WP (2015). Mouse models of rheumatoid arthritis. Vet Pathol.

[30] Weinblatt ME, Kremer JM (1988). Methotrexate in rheumatoid arthritis. J Am Acad Dermatol.

[31] Hospira Healthcare Corporation. Product monograph - methotrexate injection, USP. Reference ID: 3033070 Lake Forest, IL 60045. October, 2011.

[32] Abolmaali SS, Tamaddon AM, Dinarvand R (2013). A review of therapeutic challenges and achievements of methotrexate delivery systems for treatment of cancer and rheumatoid arthritis. Cancer Chemother Pharmacol.

[33] Bader R. The development of targeted drug delivery systems for rheumatoid arthritis treatment. In: Lemmey AB, editor. Rheumatoid Arthritis - Treatment. IntechOpen2012.

[34] Shaji J, Lal M (2013). Nanocarriers for targeting in inflammation. Asian J Pharmaceutical Clin Research.

[35] Geiger R, Aron RH, Todhunter P (2009). The climate near the ground.

[36] Hawkins P, Armstrong R, Boden T, Garside P, Knight K, Lilley E (2015). Applying refinement to the use of mice and rats in rheumatoid arthritis research. Inflammopharmacology..

[37] Thornton S, Strait RT (2016). Head-to-head comparison of protocol modifications for the generation of collagen-induced arthritis in a specific-pathogen free facility using DBA/1 mice. Biotechniques..

[38] Tanaka S, Toki T, Akimoto T, Morishita K (2013). Lipopolysaccharide accelerates collagen-induced arthritis in association with rapid and continuous production of inflammatory mediators and anti-type II collagen antibody. Microbiol Immunol.

[39] Brand DD, Latham KA, Rosloniec EF (2007). Collagen-induced arthritis. Nat Protoc.

[40] Vincelette J, Xu Y, Zhang LN, Schaefer CJ, Vergona R, Sullivan ME (2007). Gait analysis in a murine model of collagen-induced arthritis. Arthritis Res Ther..

[41] Bolon B, Stolina M, King C, Middleton S, Gasser J, Zack D (2011). Rodent preclinical models for developing novel antiarthritic molecules: comparative biology and preferred methods for evaluating efficacy. J Biomed Biotechnol.

[42] Lee JM, Langdon SE (1996). Thickness measurement of soft tissue biomaterials: a comparison of five methods. J Biomech.

[43] Pauk J, Wasilewska A, Ihnatouski M. Infrared thermography sensor for disease activity detection in rheumatoid arthritis patients. Sensors (Basel). 2019;19(16):3444. 10.3390/s19163444 PMC6720753 31394720

[44] Duricki DA, Soleman S, Moon LDF (2016). Analysis of longitudinal data from animals with missing values using SPSS. Nat Protoc.

[45] Fitts DA (2011). Ethics and animal numbers: informal analyses, uncertain sample sizes, inefficient replications, and type I errors. J Am Assoc Lab Anim Sci.

[46] Holmdahl R (2014). Studies of preclinical rheumatoid arthritis synovial histology-a comparison of animal models: comment on the article by de Hair et al. Arthritis Rheumatol.

[47] Kisten Y, Györi N, af Klint E, Rezaei H, Levitsky A, Karlsson A, et al. Detection of clinically manifest and silent synovitis in the hands and wrists by fluorescence optical imaging RMD Open 2015;1:e000106. 10.1136/rmdopen-2015-000106 PMC4612680 26535142

[48] Brenner M, Braun C, Oster M, Gulko PS (2006). Thermal signature analysis as a novel method for evaluating inflammatory arthritis activity. Ann Rheum Dis.

[49] Qi R, Majoros I, Misra AC, Koch AE, Campbell P, Marotte H (2015). Folate receptor-targeted dendrimer-methotrexate conjugate for inflammatory arthritis. J Biomed Nanotechnol.

[50] Ha YJ, Lee SM, Mun CH, Kim HJ, Bae Y, Lim JH (2020). Methotrexate-loaded multifunctional nanoparticles with near-infrared irradiation for the treatment of rheumatoid arthritis. Arthritis Res Ther.

